# A questionnaire-based validation of metacognitive strategies in writing and their predictive effects on the writing performance of English as foreign language student writers

**DOI:** 10.3389/fpsyg.2022.1071907

**Published:** 2022-12-21

**Authors:** Chenghai Qin, Ruru Zhang, Yanling Xiao

**Affiliations:** College of Foreign Languages, Hainan University, Haikou, China

**Keywords:** metacognitive knowledge, writing, metacognitive awareness, metacognitive regulation, self-regulation

## Abstract

**Introduction:**

This study—drawing upon data from a questionnaire—examined 503 Chinese university students’ metacognitive strategies in writing (MSW). The focus was on Chinese student writers who are learning English as a foreign language (EFL).

**Methods:**

The examination was conducted through a survey on MSW and a writing test administered at the end of the semester. We employed exploratory factor analysis (EFA) and confirmatory factor analysis (CFA) for data analysis. Multiple regression analysis was also adopted for understanding the predictive effects of strategies on writing performance.

**Results:**

The findings provided validity to MSW, including person, task, strategies, planning, monitoring, and evaluating. The different components of MSW were reported to significantly affect the participants’ writing performance. The findings highlight that EFL student writers were aware of metacognitive writing strategies. The MSW survey could be used to assess EFL students’ metacognitive writing strategies and develop curricula in writing strategy training.

**Conclusion:**

Writing instruction can direct learners’ ability to acquire metacognitive writing strategies, particularly those of planning, monitoring, and evaluating, to build their awareness as agents in EFL writing. Relevant pedagogical implications are discussed.

## Introduction

Metacognitive strategies are essential to the process of learning to write when learning English as a foreign language (EFL; Nguyen and Gu, 2013; Teng, 2016, 2019; Teng and Yue,2022). However, in the Chinese EFL context, for which English writing instruction typically emphasizes grammatical correctness rather than idea development, learners may find it difficult to build an awareness of using metacognitive writing strategies (Ruan, 2014). Through a mixed-methods study, Amani (2014) found that explicit metacognitive strategy instruction had a positive impact on the writing competence of L2 writing students. However, in terms of EFL writing, university EFL students may find it challenging because of their lack of awareness of metacognitive writing strategies (Teng, 2019). In addition, EFL learners in the Chinese context receive limited English language input, making it more challenging to learn to write. Student writers are expected to have repertoires of strategies when learning to write (Raimes, 1987). In particular, they need to build an advanced level of “self-initiated thoughts, feelings, and actions” for them to “attain various literary goals” (Zimmerman and Risemberg, 1997, p.76). Hence, metacognitive writing strategies are essential to possible improvements in EFL writing.

Nevertheless, even though students are taught how to plan, monitor, and evaluate their own writing, students may know little about themselves as writers (Leung and Hicks, 2014). They may also not recognize their own writing strengths or weaknesses, tending to overemphasize the latter and overlook any progress they have made or can make in their writing (Teng, 2016). Wenden (1998) argued that metacognitive knowledge is a prerequisite for self-regulation, and metacognitive knowledge is essential to learner autonomy because it “informs planning decisions taken at the outset of learning and the monitoring processes that regulate the completion of a learning task and decisions to remediate; it also provides the criteria for evaluation made once a learning task is completed” (p. 528). Teng and Zhang (2021) argued that there is a dynamic and longitudinal relationship between metacognitive knowledge and reading and writing in a foreign language context. However, teachers may not recognize the importance of metacognitive knowledge in Chinese EFL writing contexts, wherein teaching academic writing is product oriented (Teng and Zhang, 2016). The student writers were passive and found it difficult to keep positive beliefs in writing (Bruning and Horn, 2000). This may be related to learners’ lack of awareness of self-regulation in writing. They may exert more effort learning vocabulary knowledge and grammar for writing, rather than being an agent for writing (Graham and Harris, 2000). Student writers need self-awareness, motivation, and positive behavioral skills for writing (Zimmerman, 2002, p.65–66). Metacognitive writing strategies are thus essential to EFL students’ writing performance.

Self-regulation principles, measurements, and practices have a solid ground for enriching second and foreign language learning and teaching (Teng and Zhang, 2022). Through a socio-cognitive approach to writing, Nishino and Atkinson (2015) argued that writing is primarily a cognitive activity and that cognition plays a vital role in writing and its development. To help students become competent English writers and autonomous learners, instructors need to support their development of metacognitive strategies. However, scarce attention was paid to writing strategies from the perspective of metacognition, particularly for low-achieving students in the EFL context. The present study examined Chinese university EFL students’ metacognitive strategies in EFL writing. We aim for the following purposes: (a) to assess the reliability of a new scale, which we named it as metacognitive strategies in writing (MSW) and (b) to explore how different components of MSW predict EFL students’ writing performance. The findings are insightful in helping researchers and classroom practitioners to diagnose the needs of metacognitive strategies in writing and develop guidelines for instructing writing courses for university EFL students. The findings shed lights on how to teach EFL writing and deliver more effective program for writing teacher preparation.

## Literature review

### Language learning strategies

Oxford (1990) classified a list of language learning strategies based on cognitive learning theory. These strategies include memory, cognitive, compensatory, affective, social, and metacognitive strategies. Past studies have documented differences in strategy use between more and less successful learners. For example, successful learners use these strategies in larger numbers and at higher frequencies (Magogwe and Oliver, 2007). Most importantly, cognitive and metacognitive strategies are associated with a higher level of language proficiency (Peacock and Ho, 2003). However, contradictory findings were also reported, showing that less successful learners used more strategies than more successful learners did because the former automatized their language learning process (Oxford and Cohen, 1992). Another point worth noting is that unsuccessful learners may adopt a large number of strategies frequently, but it does not necessarily mean that they are able to identify appropriate strategy use. In fact, it was reported that successful learners were able to identify appropriate strategies depending on the task requirements, but unsuccessful learners failed to choose the most appropriate and efficient strategies during the task (Chamot and El-Dinary, 1999).

Although ample research has been reported relating to learners’ proficiency level and strategy use, learner variables, such as cultural background and national origin, could have a strong influence on learners’ strategy use (Oxford and Nyikos, 1989). Therefore, their findings might not be generalizable to learners with completely different cultural backgrounds. In light of this, Lai (2009) conducted a questionnaire survey that investigated the relationships between the language learning strategies used by 418 EFL learners in Taiwan based on learners’ language proficiency and their use of strategies. While the more proficient learners used metacognitive strategies and cognitive strategies most frequently and memory strategies least frequently, the less proficient learners preferred social and memory strategies to cognitive and metacognitive strategies. This finding partially echoes Wu (2008), who reported that higher-proficiency EFL students in Taiwan used learning strategies more often than lower-proficiency EFL students did, especially the cognitive, metacognitive and social strategies.

Although research documented in the literature examines general language learning strategy use, it is possible that these summarized findings could serve as a reference for the specific examination of metacognitive strategy use during English writing.

### Understanding metacognition

Metacognition is multidimensional and domain-general. When we talk about metacognition, we may need to mention the theory of mind (Flavell, 1979). Such theory is the foundation of understanding metacognition. Generally, metacognition is related to self-regulatory capacity because metacognition provides individuals with domain knowledge and regulatory skills that are essential to become an agentive learner in relevant domains (Schraw, 2001, p. 7). Metacognition refers to how learners build an awareness of their own thinking processes and executive processes (Flavell, 1979). Metacognition is essential to helping learners regulate their cognitive processes, and finally, becoming an independent thinker and learner. Zhang and Zhang (2019) applied metacognition in second and foreign language learning, and posited that EFL learners need to plan, monitor, and evaluate their cognitive processes for better language learning performance.

Metacognition includes metacognitive knowledge and metacognitive regulation. Flavell (1985) suggested that person, task, and strategy knowledge are three key elements of metacognitive knowledge. Wenden (1998) explained the three elements. For example, person knowledge is the knowledge for the learners to control their cognitive processes. Task knowledge is the knowledge that can be helpful for the learners to understand the purpose, nature, and demands of different task conditions. Strategy knowledge is the knowledge of different important strategies that are helpful for realizing the pre-determined goals. Metacognitive regulation entails three skills: planning, monitoring, and evaluating (Schraw, 1998). Planning refers to the ability to appropriately select the strategies and adequately allocate the resources for completing tasks. Monitoring refers to learners’ capacity to observe their task performance. Evaluating means learners’ capacity to reflect on their learning outcome and the use of different strategies for self-regulation.

**Figure 1:**
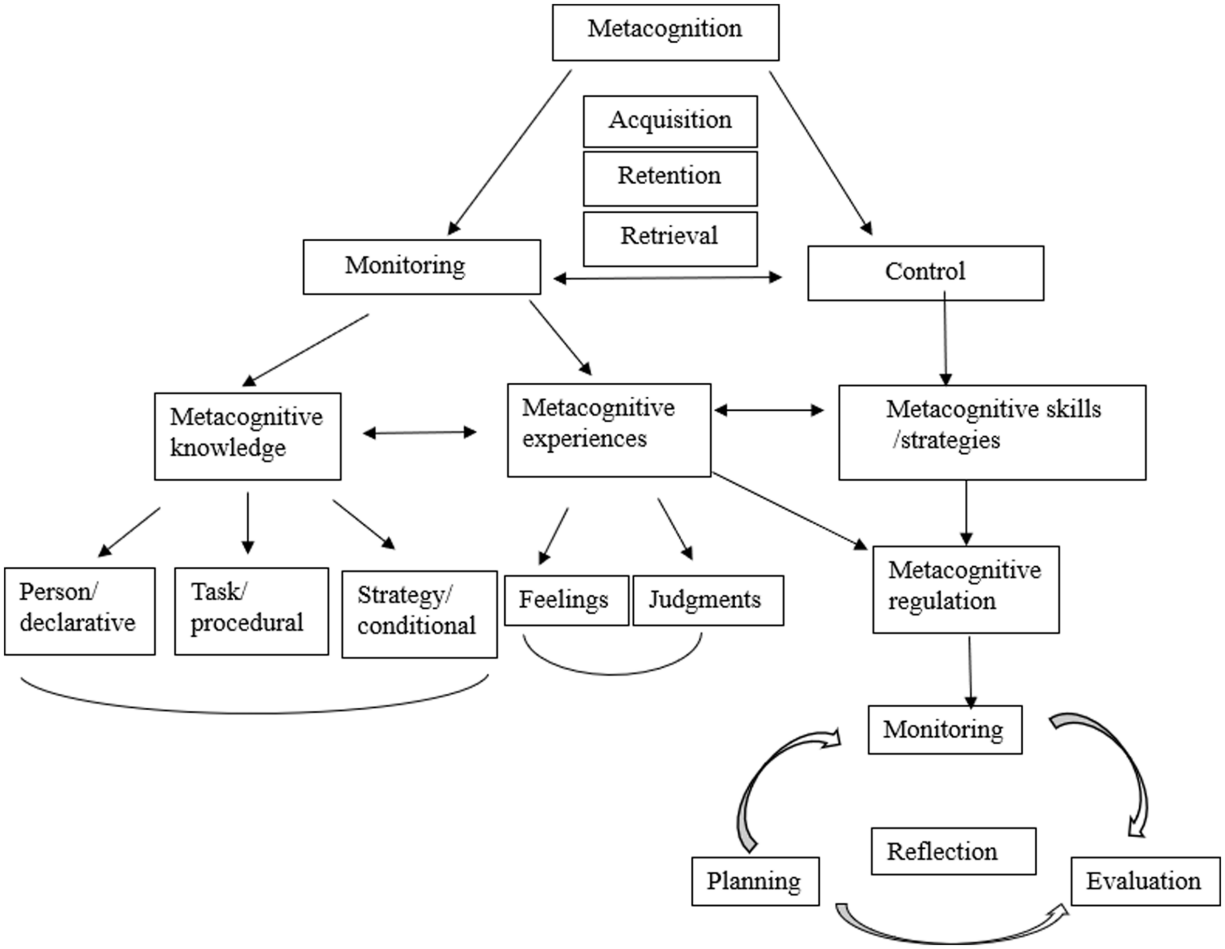
The multifaceted elements of metacognition (Teng et al., 2022, p. 171).

Teng et al. (2022) summarized the procedures of understanding metacognition. First, monitoring function and control of cognition are two important functions of metacognition. In order to realize the functions, individuals need to process three major stages, i.e., acquisition, retention, and retrieval. Second, learners need metacognitive knowledge and metacognitive experiences to process the monitoring function. In contrast, they need metacognitive strategies or metacognitive skills to fulfill the needs of control of cognition. Third, metacognitive knowledge, metacognitive experiences, and metacognitive skills are interconnected with each other. Metacognitive knowledge includes person, task, and strategies. Metacognitive experiences include feelings and judgments. Metacognitive skills are important for their metacognitive regulation, which needs learners to plan, monitor, and evaluate their learning process. Finally, reflection is the outcome of the interconnected process of planning, monitoring, and evaluating (Figure 1).

### Metacognitive strategies in EFL writing

Macaro (2010) maintains that strategic behavior plays a vital role in second language learning success and proposes that strategic behavior should be essential to linguistic knowledge resources. Dornyei (2010) emphasizes that students need a repertoire of appropriate task-related plans, scripts, and self-regulatory strategies that are activated by their ideal L2 selves; that is, learners’ aptitude, motivation, goals, and self-regulatory strategies all interact and affect one another in the SLA process. Writing strategies include rhetorical strategies, metacognitive strategies, cognitive strategies, and social/affective strategies (Wenden, 1991; Riazi, 1997). Writers explore rhetorical strategies to organize and present their ideas based on the writing conventions of the target language. Metacognitive strategies are used to monitor the writing process consciously and evaluate the effectiveness of writing actions. Cognitive strategies are used to implement actual writing actions. Social/affective strategies are employed to interact with others and to regulate emotions, motivation, and attitudes in writing.

Wenden (1991) classifies writing strategies based on metacognitive and cognitive frameworks. She distinguishes general executive metacognitive strategies of planning, self-monitoring, and self-evaluating from more specific cognitive strategies, such as clarification, retrieval, resourcing, avoidance, and verification. Each of these metacognitive strategies is discussed below.

Planning for writing involves thinking and self-questioning strategies such as identifying one’s purpose, activating background knowledge, and organizing ideas. Planning is not limited to a specific stage of writing but rather appears recursively throughout the writing process. Flower and Hayes (1981) identified three different types of planning strategies based on the focus of the goal: (1) generating ideas; (2) setting procedural goals; and (3) organizing. Generating ideas includes retrieving information from long-term memory, revising old ideas to incorporate new information, drawing inferences, making connections, and looking for examples, contradictions, and objections. Setting procedural goals includes content goals (e.g., plans for content, text structure and audience, and criteria for evaluation) and process goals (how to proceed, generated by the writer, done at any time during the composing process, followed or preceded by generating ideas, revising strategies, etc.). The third strategy (organizing) includes selecting the most useful materials produced during the generating process and organizing them in the writing plan. Organizing strategies include grouping and sequencing ideas, deciding on the presentation of the text, planning the introduction and conclusions, and structuring the text based on a particular genre. Furthermore, in using these strategies, it is essential to consider the audience, topic, and rhetorical knowledge. Planning in EFL writing determines how writers write in subsequent stages. It engages them in metacognitive activities that allow them to consider the purpose and goals for writing, identify their audience, decide upon voice, and generate a framework for their essays.

Monitoring involves conscious control and regulation of the writing process. Hayes and Flower (1980) include self-monitoring in their model of the cognitive processes of writing, noting that the ability to self-monitor the composing process is an important part of writing strategies. Charles (1990) claims that self-monitoring makes it easier for L2 students to avoid uncertainty about any part of their text, to find direct answers to their queries and to encourage them “to look critically and analytically at their writing and to place themselves in the position of readers” (p. 289). The more important functions of self-monitoring are controlling, directing, and sequencing the composing processes and one’s progress in the task. Monitoring allows the writer to decide whether something needs to be retrieved, whether new ideas need to be further generated, or whether a given subprocess has ended. Monitoring allows L2 writers to evaluate the effectiveness of writing strategies and how and when to check the outcomes of problem-solving processes and strategically regulate the processes according to cognitive goals (Mayer, 1999).

Self-evaluating—experiencing the quality of one’s writing in relation to one’s goals—is crucial for developing an individual’s perception of writing. In self-evaluation, students can recognize weaknesses, identify needs, and make changes (Zimmerman, 2002). In cognitive research, evaluation has been characterized as a strategy for considering the outcome of the undertaken task, an essential metacognitive strategy that successful learners need to execute and control.

### Empirical studies on the use of metacognitive writing strategies

Various studies have been conducted on EFL students’ use of metacognitive writing strategies. Employing think-aloud protocols and immediate retrospective interviews, Chien (2012) investigated the differences in writing strategies and English writing achievements of 20 low-achieving and 20 high-achieving student writers in Taiwan. Chien found that high-achieving student writers were more aware of and focused more on, formulating their position statements when planning, generating, revising, and editing their essays and focused more on correcting grammatical and spelling errors. Teng and Zhang (2016) validated questionnaire-based self-regulated strategies in EFL writing and highlighted planning, monitoring, and evaluating in EFL writing. Teng and Huang (2019) also suggested that learners’ self-regulated strategies in writing, as well as their English proficiency and language learning experiences, and significantly influenced their EFL writing. In a recent publication (Teng et al., 2022), two experimental studies were reported. Study 1 adopted a factorial design using exploratory and confirmatory factor analysis to validate a self-regulatory writing strategy questionnaire. Study 2 assessed the predictive effects of the different components of the scale on students’ writing performance. The results supported the construct validity for the six strategy factors, i.e., writing planning, goal-oriented monitoring, goal-oriented evaluation, emotional control, memorization, and metacognitive judgment. The factors also predicted writing performance. Zhang and Qin (2018) also validated the newly developed scale on metacognitive strategies in a multimedia writing context. The results provided evidence for the validation of planning, monitoring, and evaluating strategies. In an early empirical study on the importance of planning in EFL writing, Graham et al. (1995) examined differences between expert and less-skilled L2 writers. They found that expert L2 writers spent considerable time planning and appeared to have higher-level plans and self-conscious control of their planning. In contrast, less-skilled EFL writers were less likely to use knowledge of textual structure in planning, to use heuristic strategies in searching their memory for content, or to establish goals to direct the writing process and were more likely to engage in “knowledge telling” (i.e., writing everything they knew about a topic and stopping when they felt that they had written down everything they knew). Less-skilled writers did not write with goals or plans in mind; rather, they tended to generate ideas through free writing and usually did not organize those ideas. As shown in a longitudinal study (Teng and Zhang, 2021), learners’ L2 writing development was dependent on their initial level of metacognitive knowledge. This is evidence for the strong correlation between metacognitive knowledge and writing.

Nguyen and Gu (2013) explored the impact of strategy-based instruction on promoting learner autonomy (operationally defined as learner self-initiation and learner self-regulation) of students at a Vietnamese university; 37 students were in an experimental group, and 54 students were in two control groups. After an 8-week metacognition training intervention, students in the experimental group were found to have improved their planning, monitoring, and evaluating of a writing task more than those in the two control groups. The findings suggest that strategy-based instruction on task-specific metacognitive self-regulation improves learner autonomy and writing performance. Teng (2020) also incorporated training of metacognitive strategies for EFL learners. There were two groups of learners, i.e., those with group feedback guidance and those with self-explanation guidance. The results supported the positive effects of group metacognitive support on EFL students’ writing. EFL students need to build a certain level of metacognitive awareness to manage themselves as writers.

Bai et al. (2014) conducted a questionnaire survey to explore the relationship between 1,618 Singapore primary school pupils’ reported use of strategies in learning to write and the correlation with their English language proficiency. They found that participants used a wide range of writing strategies at medium frequency. They also reported a significant correlation between the participants’ English language proficiency and the use of writing strategies such as planning, text-generating, revising, monitoring and evaluating, and resourcing. Similar results were also found in Bai and Guo (2021), wherein high achievers reported higher levels of motivation (i.e., growth mindset, self-efficacy, and interest) and self-regulated learning strategy use than the average achievers, and average achievers reported more strategy use than the low achievers, Ma and Teng (2021) collected qualitative data from two undergraduate university students learning English as L2 in Hong Kong to explore their use of writing strategies. They reported that both students realized the importance of self-evaluation and revision. It seems that the students perceived affordances in the kind of writing that enabled them to play an active role in seeking, interpreting, and using teacher feedback to perform the evaluation and modification of their own work. However, variations in engagement in the process of learning to write and their metacognitive knowledge development were also detected. For example, students’ varying degrees of engagement may result in various degrees of developing metacognitive awareness. Teng et al. (2022) validated a new instrument, i.e., the Metacognitive Academic Writing Strategies Questionnaire (MAWSQ). Analyses were conducted through a series of Confirmatory factor analyses (CFA). Results supported two hypothesized models, i.e., an eight-factor correlated model and a one-factor second-order model. Model comparisons supported the role of metacognition as a higher-order construct. Metacognition also explains the eight metacognitive strategies, including declarative knowledge, procedural knowledge, conditional knowledge, planning, monitoring, evaluating, information management, and debugging strategies. Those strategies also significantly influenced EFL writing performance.

Overall, the studies on metacognition development reviewed in this section highlight the importance of the high-level cognitive processes involved in composing, the development of the autonomous and self-regulated use of effective writing strategies, and the formation of positive attitudes about writing. Metacognitively oriented learners are aware of both their own learner characteristics and the writing task and are able to select, employ, monitor, and evaluate their use of metacognitive strategies.

## The present study

Metacognition functions as an important predictor in EFL writing performance. We aim for two purposes in the present study. First, we attempted to validate a questionnaire on metacognitive strategies in writing. Second, we assessed the predictive effects of different metacognitive strategies in the outcome EFL writing. The present study sheds light on learners’ awareness and use of metacognitive writing strategies. The present study includes two questions:

1. What is the evidence to support the validity and reliability of metacognitive strategies in writing?
2. What is the evidence for the predictive effects of metacognitive strategies on EFL writing proficiency?

## Materials and methods

### Participants

The present study included 503 participants. They were undergraduate students at a university in China. They were first-year students with Chinese as their first language and English as a foreign language. They had received at least 6 years of formal English instruction. Writing is a subject to be taught in college English and a compulsory course for all the participants. We selected the participants because they were all enrolled in a university English course. The first author was teaching the participants, and the sample of participants was a convenient sample. Among the 503 students, 351 were men and 152 were women. An unequal gender balance may be because most of the students were from science and engineering majors. Originally, there were 700 students who responded to the questionnaire. We finally selected data from 503 students for data analysis. Some participants’ data were excluded because of missing values or because some were unable to take the writing test. They attended the study voluntarily by signing the consent form.

### Questionnaire development

The questionnaire, which was named Metacognitive Strategies in Writing (MSW), was developed through item generation, reference consultation, initial piloting, psychometric evaluation, and exploratory factor analysis (EFA) in a pilot study. We first invited 10 students to reflect on their writing practices and strategies. The students were mainly interviewed about the strategies they adopted for writing. We generated approximately 50 items based on analyzing the transcriptions of learners’ interviews. In the next stage, we consulted relevant literature on metacognition, self-regulation, and language learning strategies (Schraw and Dennison, 1994; Oxford, 2013; Teng et al., 2022). We selected the items that fit with metacognition theories. In the third stage, we invited the 10 students to check the items. In the fourth stage, which was psychometric evaluation, we invited two researchers in L2 writing to assess the items. Based on the comments, we finally removed 10 items. In the final stage, we ran an EFA with a sample of 360 students with similar backgrounds. We deleted 10 items with unsatisfactory factor loading values. The final questionnaire includes 30 items, which are in the Appendix.

This questionnaire was a novel one as it was based on metacognition theory, through which the focus was on understanding metacognitive knowledge and regulation in learning to write. We adopted a seven-point Likert scale (i.e., from 1, Strongly disagree to 7, Strongly agree). MSW focuses on metacognitive knowledge and metacognitive regulation. Metacognitive knowledge includes three factors, i.e., person, task, and strategies. Metacognitive regulation includes three factors: planning, monitoring, and evaluating. Cronbach’s alpha, which ranged from 0.81 to 0.90 for the six factors, ensured the internal consistency of responses to the items. The questionnaires were administered to the participants in Chinese. The author translated into Chinese while a research assistant was invited to check the translated items through back translation.

### Writing test

A writing test from IELTS (writing task 2) was adopted to measure learners’ writing proficiency. Students were required to write at least 250 words within 1 h. Students were asked to respond to the topic provided by giving and justifying an opinion, discussing the topic, summarizing details, outlining problems, identifying possible solutions and supporting what they wrote with reasons, arguments and relevant examples. The topic proposed the possible influence of social media sites on personal relationships.

The marking scheme was consistent with the writing rubrics in IELTS. However, we adjusted it to fit with our school assessment needs. Each learner was awarded with six marks for task response, coherence and cohesion, lexical resource, and grammatical range and accuracy. The maximum possible score was 24 points. A total of 40 English teachers were paid to rate the writing. The teachers did not know the participants’ identities. They also joined a training session on the marking scheme. Disagreements on marking were subject to further discussion. The Cronbach’s alpha for the test was.85, indicating acceptable reliability.

### Procedures

We invited 20 EFL teachers to help us distribute a QR code to the students through WeChat group. The students spent an average of 6 min completing the questionnaire. The writing test was administered as an exercise for all students during class. They needed to complete it within 1 h. The format for the writing test was a paper-and-pencil format. All participants received the same format for the questionnaire and the writing test.

### Data analysis

The final dataset was run through a series of confirmatory factor analyses (CFAs). STATA was used for data analysis. CFA is used to test a theoretical model by confirming factors, correlations, covariance patterns, and residual or error values within a data matrix (Byrne, 2016). We used the maximum likelihood (ML) estimation method. The model fit was evaluated through the following statistics: a chi-square statistic, the degrees of freedom (df), *p* value, the ratio of chi-square χ^2^ divided by the df, the root mean square error of approximation (RMSEA), the standardized root mean square residual (SRMR), the comparative fit index (CFI), and the Tucker–Lewis Index (TLI; DiStefano and Hess, 2005). The following criteria are a relatively good fit between the hypothesized model and the observed data: the value of RMSEA should be close to 0.06, the value of SRMR should be close to 0.08, and the values for CFI and TLI should be close to 0.95 (Hu and Bentler, 1999). Finally, multiple regression analysis was adopted to evaluate the predictive effects of MSW on students’ writing proficiency.

## Results

### Descriptive statistics

The kurtosis and skewness values for the metacognitive strategies in writing, as well as the mean and standard deviation, are shown in Table 1. The means of the six factors ranged from 3.346 to 4.079, with the two factors, monitoring and evaluating, greater than 4. There were no noticeable variations based on the standard deviation values.

**Table 1:** Means, standard deviations. and normality test.

Strategy category	Mean	S. D.	Skewness	Kurtosis
Person	3.346	1.014	0.914	0.182
Task	3.620	0.782	0.225	−0.666
Strategy	3.762	0.786	0.513	−0.033
Planning	3.976	0.931	0.367	−0.822
Monitoring	4.075	0.841	0.359	−0.633
Evaluating	4.079	0.840	0.330	−0.516

### Exploratory factor analysis in the pilot study

Exploratory factor analysis was conducted on a sample of 360 learners from similar background in the pilot study. We examined the adequacy of the sample. The Kaiser-Meyer-Olkin value was 0.914, which appropriate for EFA (Tabachnick and Fidell, 2001). Bartlett’s test of sphericity was significant, *p < 0*.001; thus, the matrix was adequate for factor analysis. We adopted principal component analysis as a factor extraction method. We finally extracted six factors that explained 57.411% of the variance (Table 2). The scree plot showed a considerable drop after the sixth factor, for which we excluded other possible factors. Based on key theories in metacognition, we named the six factors as following: person, task, strategies, planning, monitoring, and evaluating.

**Table 2:** Extraction results for the six factors.

Factors	Eigen value (Rotated)	% of Variance (Rotated)	Cumulative % of Variance (Rotated)
1	5.023	11.232	13.202
2	5.002	11.342	21.217
3	4.532	10.543	32.832
4	3.732	9.643	41.322
5	3.122	7.243	47.655
6	2.933	6.821	57.411

The six factors’ eigenvalues exceeded 1. The next step was to examine the factor loadings. We deleted 10 items with factor loadings lower than 0.4. The final version included 30 items across six factors (Table 3). Items’ factor loadings ranged from 0.534 to 0.772, while communality ranged from 0.531 to 0.754. The items hence fit their respective factors well.

**Table 3:** Results on factor loadings and the communality.

Items	Person	Task	Strategies	Planning	Monitoring	Evaluating	Communality
18	0.622						0.571
4	0.632						0.692
5	0.622						0.621
19	0.511						0.632
1	0.522						0.522
15	0.513						0.592
6		0.523					0.632
11		0.532					0.692
9		0.523					0.536
24		0.623					0.665
22		0.542					0.534
27		0.643					0.634
38			0.523				0.534
36			0.611				0.534
28			0.612				0.643
29			0.645				0.794
21			0.564				0.743
8			0.711				0.734
35				0.611			0.634
37				0.622			0.663
33				0.564			0.525
31				0.543			0.531
32					0.634		0.623
7					0.632		0.623
3					0.503		0.623
34					0.532		0.636
2						0.612	0.662
26						0.732	0.742
13						0.602	0.623
30						0.732	0.772

### Construct validity of metacognitive strategies in writing through CFA

The data fitness metrics for metacognitive strategies in writing are displayed in Table 4. Table 4 shows that the RMSEA was 0.073, less than 0.08, indicating a good fit; CFI, TLI, CNFI, IFI, and GFI all exceeded 0.9, which was ideal for adaptability. Although the χ^2^/df was 7.916, larger than 3, the scale on metacognitive strategies in writing still showed reliability when taken as a whole.

**Table 4:** Model fit indices for metacognitive writing strategies.

χ^2^/df	RMSEA	CFI	TLI	NFI	IFI	GFI
7.916	0.073	0.946	0.909	0.939	0.946	0.957

**Figure 2:**
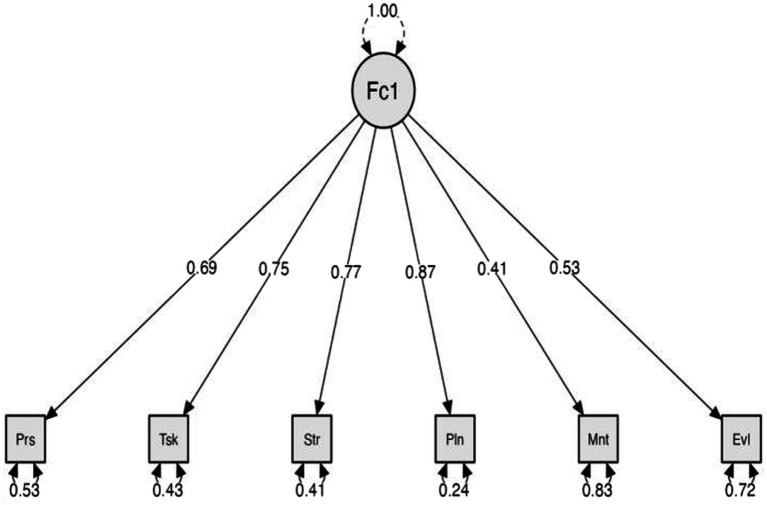
A first-order model of metacognitive strategies in writing. Prs, Person; Tsk, Task; Str, Strategy; Pln, Planning; Mnt, Monitoring; and Evl, Evaluating.

According to Figure 2 and Table 5, the factor loadings for Person, Task, Strategy, Planning and Evaluating were all greater than 0.5, while Monitoring was 0.41. Additionally, the average variance extracted (AVE) for each variable was 0.47, and the model’s convergent validity was good, as evidenced by the composite reliability (CR) being 0.84, indicating that the model had satisfactory convergent validity.

**Table 5:** Convergent validity of the model.

Path			Estimate	AVE	CR
Person	<−--	F1	0.53	0.47	0.84
Task	<−--	F1	0.41		
Strategy	<−--	F1	0.87		
Planning	<−--	F1	0.77		
Monitoring	<−--	F1	0.75		
Evaluating	<−--	F1	0.69		

### Predictive effect of metacognitive strategies in writing on EFL writing

**Figure 3:**
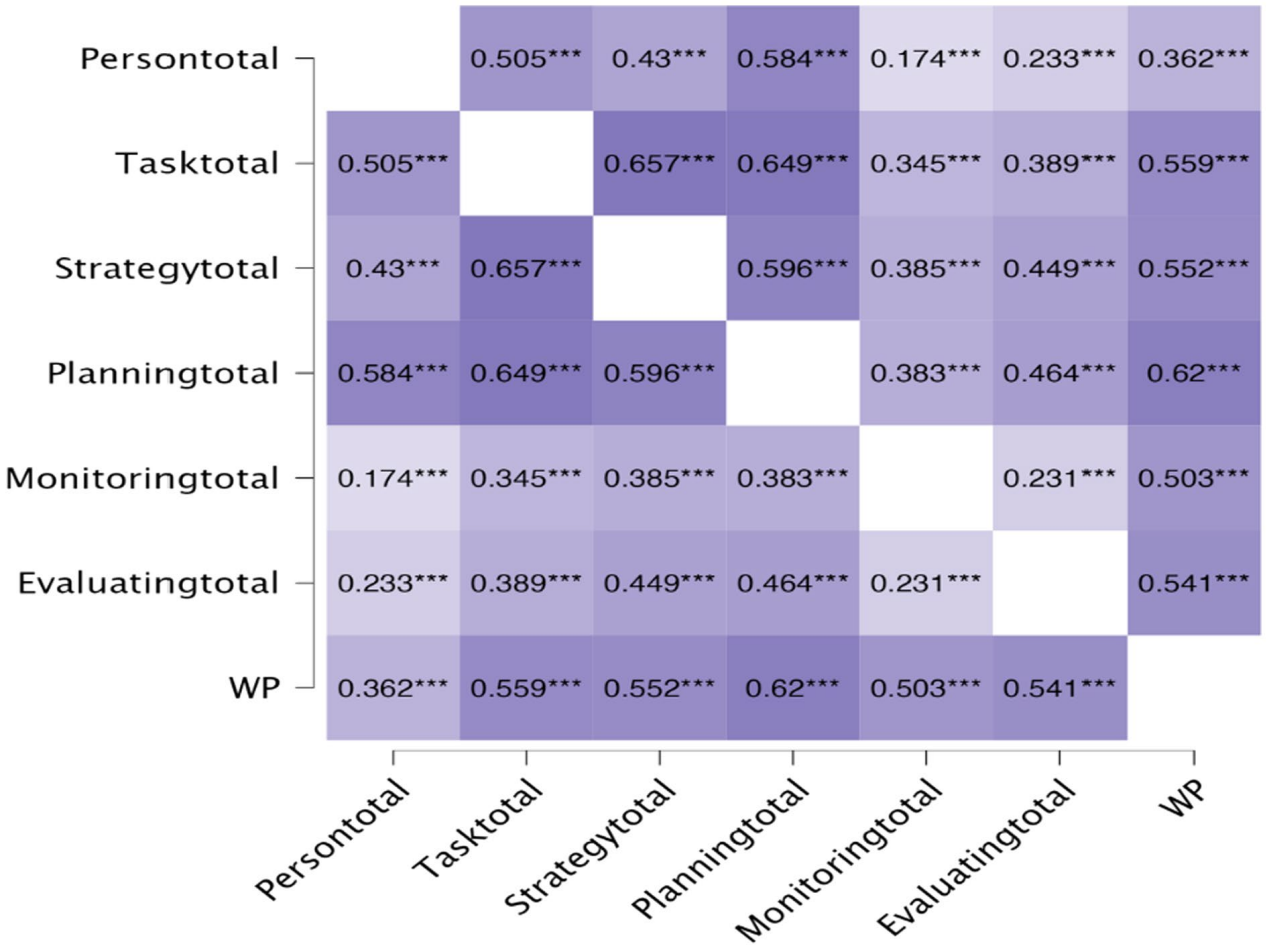
Spearman correlation for metacognitive writing strategies and L2 learners’ proficiency in English. Persontotal, Person; Tasktotal, Task; Strategytotal, Strategy; Planningtotal, Planning; Monitoringtotal, Monitoring; and Evaluatingtotal, Evaluating.

Figure 3 presents the correlations between metacognitive strategies in writing and L2 learners’ writing proficiency in English. The findings indicated that each of the six metacognitive strategies was significantly correlated with learners’ English writing performance. Writing performance (WP) was correlated with Person (*r* = 0.264), Task (*r* = 0.500), Planning (*r* = 0.584), and Monitoring (*r* = 0.408). Strategy (*r* = 0.470) and Evaluating (*r* = 0.470) were significantly correlated with WP.

**Figure 4:**
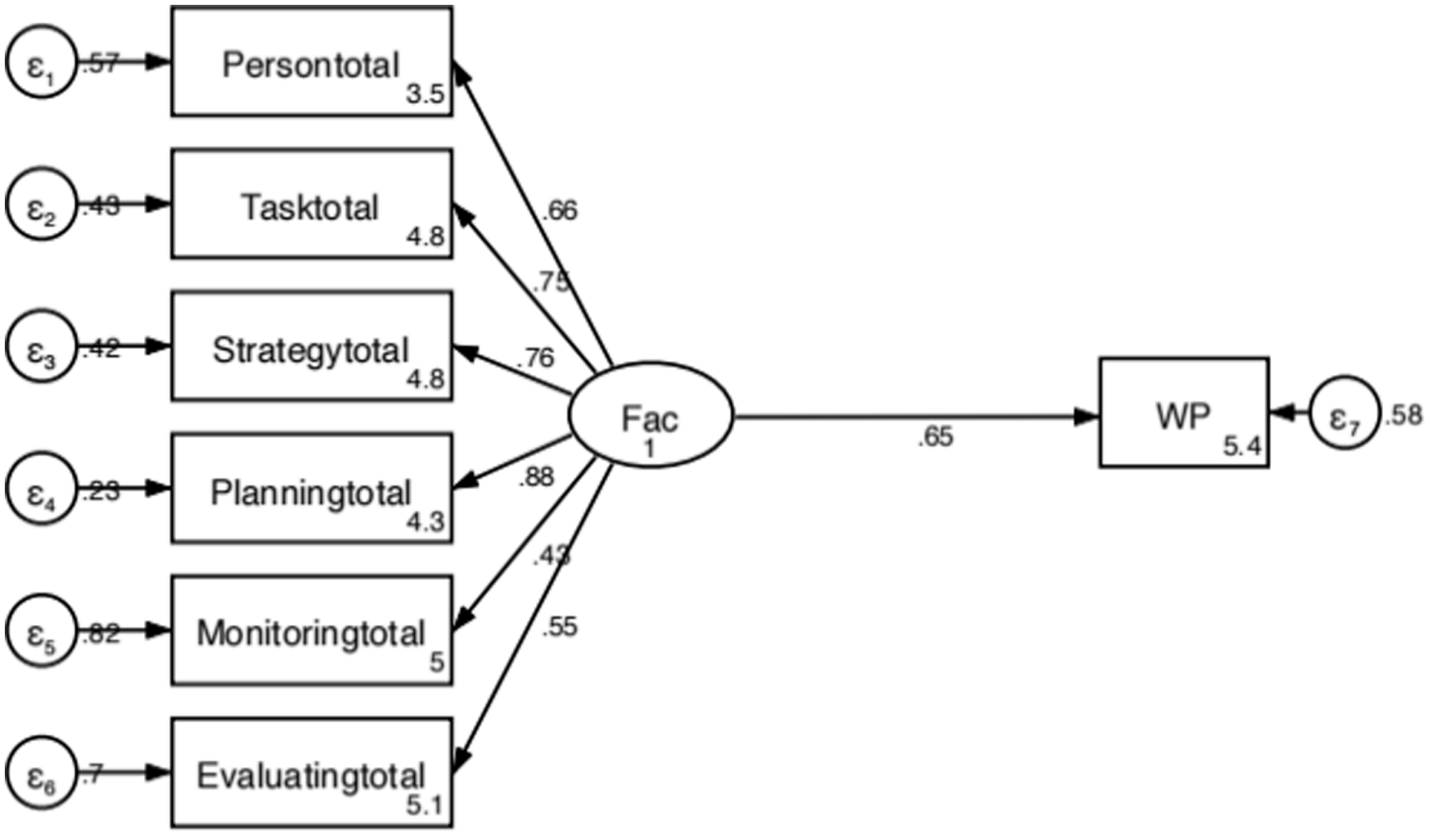
The structural equation model of metacognitive strategies in writing proficiency.

Moreover, we adopted a structural equation model to investigate the degree to which metacognitive strategies in writing predicted learners’ L2 writing proficiency. Table 6 presents the model fitness indices. For our model, seven indices (i.e., χ^2^/df, RMSEA, CFI, TLI, NFI, WIFI, and GFI) indicated acceptable model fit (Table 6). Figure 4 shows a structural equation model of the relationship between metacognitive strategies in writing and writing proficiency. The six variables on the left side of the model represent the six factors of metacognitive strategies in writing. The only rectangular variable on the right side of the model was EFL learners’ writing proficiency. The findings demonstrated that metacognitive strategies in writing had a predictive power of 0.65 for L2 learners’ writing proficiency, indicating that it could account for 65% of the variances in writing performance.

**Table 6:** Model fit indices for metacognitive writing strategies on writing performance.

χ^2^/df	RMSEA	CFI	TLI	NFI	IFI	GFI
4.154	0.065	0.951	0.923	0.931	0.945	0.953

**Figure 5:**
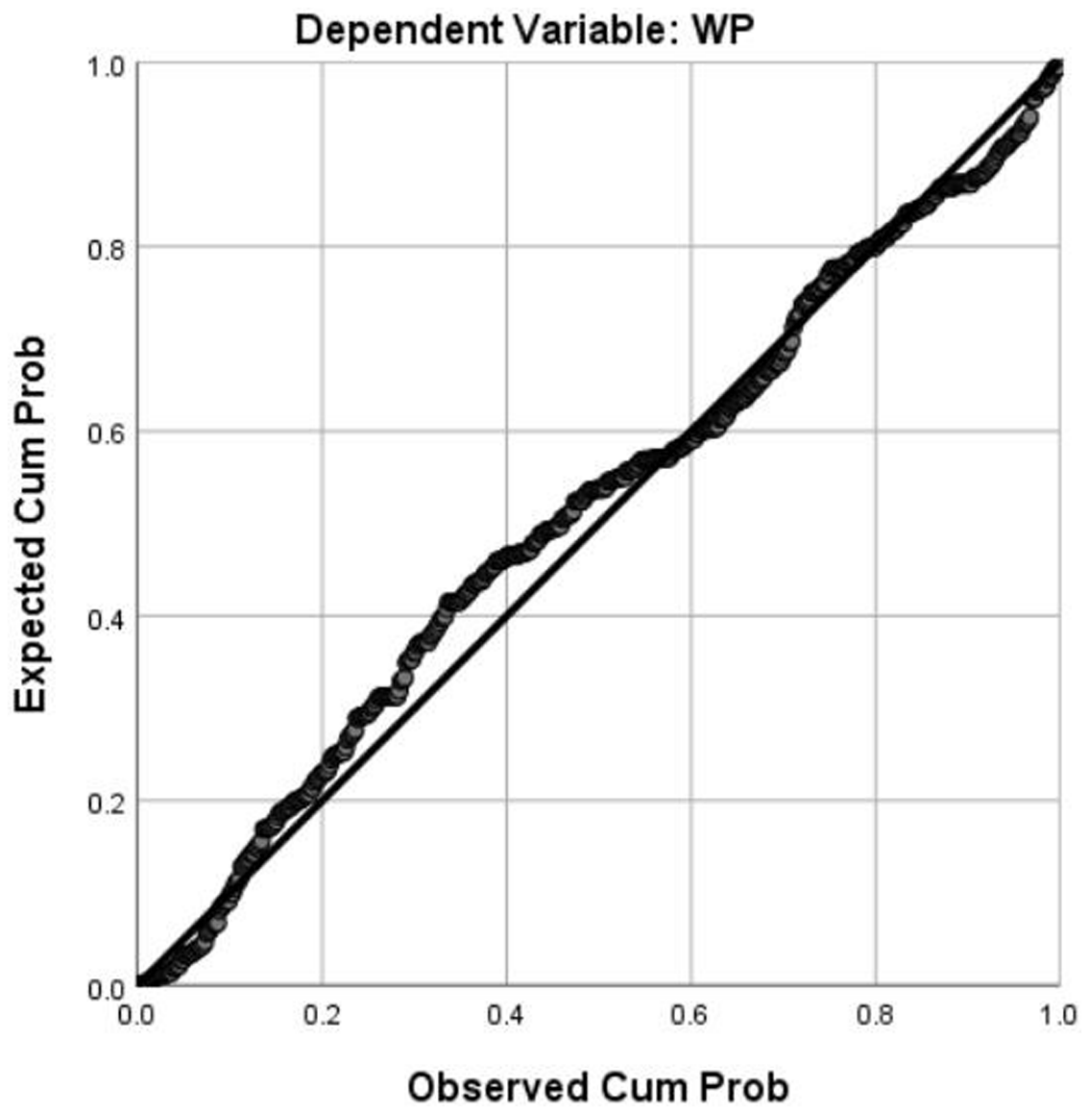
Normal P–P plot of regression standardized residual.

Regression analysis was employed in the study to show the extent to which each factor impacts writing performance. The results presented in Table 7 demonstrate that all factors significantly predicted writing competence (*p* < 0.001), with the exception of Strategy (*p* = 0.344). Planning had the greatest effect on writing abilities, and Task had the least effect. Notably, monitoring and evaluating also had a great effect on EFL learners’ writing proficiency. According to the findings, there was no multicollinearity among the strategies, as indicated by the variance inflation factor (VIF), which was less than 3. In addition, the residuals adhered to a normal distribution, as shown in Figure 5. This offered a trustworthy foundation for the regression analysis results.

**Table 7:** Linear regression results.

	Unstandardized coefficients		Standardized coefficients					Collinearity statistics	
	*B*	SE	Beta	*t*	*p*	*R* ^2^	Adjusted *R*^2^	Tolerance	VIF
(Intercept)	3.203	0.636		5.038	< 0.001	0.473	0.467		
Person	−0.112	0.024	−0.204	−4.663	< 0.001			0.555	1.803
Task	0.114	0.033	0.161	3.472	< 0.001			0.493	2.028
Strategy	0.031	0.033	0.045	0.947	0.344			0.471	2.124
Planning	0.225	0.032	0.387	7.095	< 0.001			0.356	2.807
Monitoring	0.136	0.024	0.203	5.588	< 0.001			0.802	1.247
Evaluating	0.167	0.026	0.247	6.434	< 0.001			0.721	1.387

## Discussion and conclusion

Overall, the present study aims to answer two research questions. The first research question entails the validation of a newly developed scale, which we named Metacognitive Strategies in Writing (MSW). The scale was developed based on metacognition theory. The findings supported the factorial structure of the scale. The second research question aims to answer the predictive effects of different factors of MSW in writing performance. Overall, the findings provided evidence for the factorial structure of MSW. The findings also suggested the predictive effects of different factors on writing performance.

### Validation of MSW

First, MSW is with satisfactory psychometric properties. The six factors were reliable in terms of conceptual and empirical evidence. The six factors were distinct but correlated with each other. Consistent with previous studies (Teng et al., 2022), metacognition is an important construct that can explain the significant correlations of different lower-order metacognitive dimensions in writing. In line with Schraw and Moshman (1995), metacognition is a domain that can explain self-regulatory capacity. The present study thus provides insights into metacognition theory, which can entail person, task, strategies, planning, monitoring, and evaluating (Schraw and Dennison, 1994). These strategies are interconnected and reflect the metacognitive process in writing. To build metacognitive awareness, learners need to be engaged in self-reflection and controlling of cognition (Paris and Winograd, 1990). In terms of writing, student writers need to assess their knowledge states and executive abilities to orchestrate different dimensions of metacognitive awareness. Overall, the sum of the six strategies in writing indicates EFL student writers’ overall level of metacognitive awareness in writing.

The six factors were interpreted through metacognitive knowledge and regulation. The two paradigms were also conceptualized in early studies (Flavell, 1979; Schraw, 1998; Wenden, 1998). In the present study, the two paradigms can represent key elements of metacognition. Person, task, and strategies represent learners’ beliefs and knowledge about themselves. Planning, monitoring, and evaluating reflect the process of cultivating one’ self-regulatory capacity for learning to write (Teng and Zhang, 2016; Teng et al., 2022). The findings showed a positive and significant relationship between metacognitive knowledge and regulation (Pugalee, 2001; Teng, 2016). We may need to reconsider the strong connection between metacognitive knowledge and regulation. The positive correlation may reflect the need of both knowledge and regulation in learning to write. For example, EFL students may need cognitive, metacognitive, and regulatory skills and strategies for writing (Teng, 2020). The importance of metacognitive knowledge and regulation may reflect the argument by Wolters (1999) that learners’ engagement, effort, and achievement are influenced by their metacognitive knowledge and regulation. Hence, metacognition is essential to the development of self-regulated capacity (Efklides, 2008), build identity as a student writer (Zimmerman and Risemberg, 1997, p.76), and develop self-awareness in processing their second and foreign language learning (Zhang and Zhang, 2019).

Overall, the MSW data suggest that the student writers adopted metacognitive knowledge, i.e., person, task, and strategies, to understand their strengths and weakness in writing, demands in writing, and solutions for solving problems in writing. The data also suggest that the planning strategy should be used. In the planning stage, the student writers directed their attention to fulfilling the goal of the task, planning thoroughly, evaluating the relevance and effectiveness of ideas, and eliminating inappropriate examples. Data regarding the second subscale (monitoring) reflected that students tended to use some metacognitive monitoring strategies. During the monitoring stage, the student writers focused on the overall essay development, concentrating on expanding and developing their initial ideas, evaluating their essay for clear development and focus/unity, and ignoring interruptions posed by language constraints, such as grammar and vocabulary. For the third subscale (self-evaluating), student writers tended to use certain metacognitive strategies. Student writers prioritized their attention to evaluating the unity and effectiveness of their writing before editing local errors, such as grammar, vocabulary, mechanics, and sentence variation.

### Predictive effects of metacognitive strategies in writing

The findings suggest the predictive effects of metacognitive strategies in writing. The results confirmed that the metacognitive strategies significantly predicted learners’ writing performance, which was consistent with previous studies (Teng and Huang, 2019; Teng et al., 2022). One reason is that student writers’ meager metacognitive knowledge base could result in unsatisfactory cognitive monitoring of production and progress toward the writing task goal, which, in turn, may also affect their writing performance (Teng et al., 2022). For example, lower-level writers tended to be bound to the local areas of writing, focusing on language correctness, while higher-level writers tended to focus on developing ideas and revising at the discourse level, saving editing until later (Teng and Huang, 2019). As supported in previous studies (Chien, 2012; Bai et al., 2014), higher level student writers were more aware of metacognitive strategies and used them more frequently in writing.

The argument revealed, at least for this particular sample and the chosen test, a strong and significant link between the writing abilities of EFL students and the factors of person, task, strategy, planning, monitoring, and evaluation. The EFL learners’ writing performance variations were accounted for by the six metacognitive components. The findings complement cognitive writing model of Flower and Hayes (1981), which recognizes the abilities in process writing such as planning, monitoring, and reviewing. Writing necessitates the adaptive use of emotional strategies, performance strategies, and cognitive strategies (Teng et al., 2022). The effectiveness of the strategies highlights the personal, behavioral, and environmental impacts on the regulatory capacity in learning to write (Zimmerman and Risemberg, 1997).

In our study, person and task significantly predicted writing performance with a large effect size. According to earlier research (Brown, 1987; Schraw, 2001), learners who have declarative, procedural, and conditional knowledge are more likely to become strategic learners. These results provide evidence for the idea that to master writing, EFL learners need to be able to distinguish among the various strategies, employ the appropriate strategies, and apply these strategies in their writing. The results also support earlier research that metacognitive knowledge is crucial for encouraging active involvement in applying their understanding of the writing process, recognizing the kinds of strategies useful in the growth of writing, and improving students’ writing outputs (Ruan, 2014).

In terms of metacognitive regulation, planning, monitoring, and evaluating are also important for writing performance. The effect size was quite large in the current study, for which we can detect similar results in previous studies (Teng, 2019; Teng et al., 2022). The writing abilities of students who were more self-controlled in their writing were higher in terms of goal setting, time management, and planning for writing resources (Teng and Zhang, 2016). We argue that Chinese EFL students need an awareness of planning ahead and monitoring and evaluating their planning tactics to produce successful written essays. The success of EFL academic writing depends heavily on this method. Academic writing development may be seen as a complex process for student writers because it depends on how strategically they seek information and modify their planning techniques. Students who have prepared well for academic writing are typically those who have a high level of metacognitive awareness of their writing-related objectives (Zhang and Qin, 2018). When composing their essays, lower-level writers often experienced difficulty in transferring ideas to paper during the planning, monitoring, and self-evaluating stages. The constraints in the lower-level writers’ knowledge system, including their limited linguistic competence (grammar and vocabulary), their confusion about their role as writers, their lack of knowledge strategies for overcoming writing difficulties, and their lack of knowledge of how and when to apply those strategies, impeded their composition of a meaningful essay. Consequently, many students tended to simultaneously engage in a few different stages of writing—planning, composing, revising, and editing—without any extra attention resources to monitor the overall unity and coherence of the essay, thus making the essay messy and confusing.

### Limitations and implications

Despite the positive findings, we still need to acknowledge some limitations of this study. First, the strategies described in the questionnaire were still scarce, although we showed excellent content validity. Due to the limited amount of time the learners could invest in data collection, we did not assess metacognitive experiences, another crucial component of metacognition. Interview data with students were not conducted to yield adequate methods connected to metacognitive experiences. Second, a self-report questionnaire served as the foundation for this study. Because they are dependent on the use of self-reported information, surveys may not fully reflect learners’ actual metacognitive awareness and activities. The quantitative data in future studies should be triangulated with interview data. Third, the writing test should include additional activity categories that can gauge various writing abilities. We only used one writing performance indicator. The performance of student writers may also be impacted by individual characteristics, including their language learning experiences and English proficiency level (Teng and Huang, 2019). Future studies might look at learners’ individual differences and their use of different metacognitive strategies.

However, there are also some implications based on the findings. Our findings suggest directions for pedagogy as well as future research. Considerations include issues of focus on form, development of metacognitive awareness to support metacognitive knowledge and strategies, and appreciation of the many aspects of metacognitive awareness that good L2 writing entails.

Data collected from the surveys suggest a strong connection between EFL student writers’ metacognitive knowledge and the regulation strategies they employ. Helping students become more aware of themselves as writers and the metacognitive resources upon which they can draw during the writing process may help them develop their writing competence. Language teachers and instructors should clearly instruct the importance of metacognitive strategies for EFL student writers. Related to this, metacognitive training should help students develop such awareness in learning to write. However, an important step in developing productive pedagogy for metacognitive training is assessing learners’ needs and understandings of their metacognitive strategies. The MSW might potentially contribute to EFL writing assessment in China. The MSW monitoring subscale identified the important first step in writing—planning—as a potential problem. So far as these Chinese EFL non-English major student writers were concerned, regardless of their level of English class or their majors, it seems that many of them may need to faster a metacognitive awareness. As a result, it might be helpful to provide these students with additional lessons on metacognitive strategies to address their concerns and the problems evident in their English writing. While dealing with grammatical errors is essential to writing instruction, the students should focus not only on identifying the errors and fixing them but also on finding out why they make those mistakes and how to avoid making them again. In other words, instead of correcting the errors, they should also develop their awareness of metacognitive strategies to improve their overall language competence. The instructors may also explicitly teach and demonstrate effective strategies to enhance vocabulary acquisition, such as making learners aware of lexical morphology (including word roots and suffixes), synonyms, antonyms, word categories, and similar spellings.

Clearly, it should not be assumed that learners who do not score high on norm-referenced assessments of their L2 writing need to focus exclusively on their metacognitive strategies, even though that is where they may think they need to work. Rather, these learners need to consider not only metacognitive strategies but also discourse organization and considerations of audience, voice, and genre (Hyland, 2007). It is only through an approach raising their awareness of the various aspects that contribute to good writing and through work on writing and revision strategies that they will progress optimally. Additionally, to implement these recommendations for pedagogy, teachers themselves must have substantial knowledge, professional development, and practice regarding approaches to support L2 writing. In the Chinese context, knowledge must be processed and understood in light of the metacognition and experiences of students, colleagues, and the community.

## Data availability statement

The original contributions presented in the study are included in the article/Supplementary material, further inquiries can be directed to the corresponding author.

## Ethics statement

The studies involving human participants were reviewed and approved by Hainan University. The patients/participants provided their written informed consent to participate in this study.

## Author contributions

CQ: Coordinated the study, drafted, and revised the manuscript. RZ: Data collection, drafted literature review. YX: Participated in the design of the study, revised the manuscript and performed the statistical analysis and data interpretation. All authors proofread and approved the final manuscript.

## Funding

This article is supported by the Project from the Education Department of Hainan Province, Project number: Hnky2020ZD-9.

## Conflict of interest

The authors declare that the research was conducted in the absence of any commercial or financial relationships that could be construed as a potential conflict of interest.

## Publisher’s note

All claims expressed in this article are solely those of the authors and do not necessarily represent those of their affiliated organizations, or those of the publisher, the editors and the reviewers. Any product that may be evaluated in this article, or claim that may be made by its manufacturer, is not guaranteed or endorsed by the publisher.
