# Supplementary material for: A questionnaire-based validation of metacognitive strategies in writing and their predictive effects on the writing performance of English as foreign language student writers
Source: Front Psychol. 2022 Dec 21;13:1071907. doi: 10.3389/fpsyg.2022.1071907 (PMC9812580; doi:10.3389/fpsyg.2022.1071907)
Supplement: Supplementary file 1 [file Data_Sheet_1.pdf]

Appendix:Metacognitive strategies in writing (MSW)

Instructions:
Please describe each of these statements on a scale going from 1. 'Strongly disagree', 2. 'Disagree', 3. 'More or less disagree' 4. 'Undecided' 5. More or less agree', 6. 'Agree', 7 ‘Strongly agree’

Section I Metacognitive knowledge (MK)

Person

1. I am very interested in English writing.
2. I find English writing very easy.
3. I am quite aware of my English writing competence.
4. I am quite aware of how much I know about English writing.
5. I am well aware of some grading rubrics of English expository essays, e.g., the CET-4 essay.

Task
1. I think it is very important to understand the requirements of a writing task.
2. I usually set up my writing goals based on the requirements of a writing task.
3. I write according to the genre (e.g., an exposition, a letter, a note) of an essay.
4. I use my knowledge of English discourse (e.g., thesis statement, topic sentences, coherence, transitional words) to complete a writing task.
5. I have a strong awareness of my readers and my communication with them.

Strategy

1. I usually think in English when I write English essays.
2. I tackle the problems that I encounter during the writing process with appropriate remedial strategies.
3. My composing process is usually like this: Analyzing the writing prompt, generating ideas, planning, outlining, drafting, and revising.
4. Based on the situation, I would repeat one or more of the above writing steps to revise my essay.
5. When writing, I pay more attention to the content development of my essay.

Section II Metacognitive regulation (MR)

Planning
1. Before writing, I pay attention to the genre (e.g., an exposition, a letter, a note) of a writing task.

2. Before writing, I pay attention to the purpose (e.g., to express opinion, to thank someone, to complain about a service) of a writing task.
3. Before writing, I pay attention to the audience (e.g., the instructor, customers, supervisor, classmates, general public...etc.) of a writing task.
4. Before writing, I pay attention to specific parts of the language (e.g., the wording and grammar) of a writing task.
5. Before writing, I consciously recall the model essays related to the writing prompt.

Monitoring
1. When writing, I check if my essay meets the requirements of the writing task.
2. When writing, I check if the content of my essay is relevant to the topic.
3. When writing, I check if the organization of my essay is logical.
4. When writing, I check if the language of my essay is clear enough.
5. When writing, I check if my word usage is accurate enough.

Evaluating
1. After finishing writing, I reflect on the content of my essay to see if any addition or deletion is needed.
2. After finishing writing, I reflect on the accuracy of my grammar.
3. After finishing writing, I reflect on the organization of my essay to see if it is clear enough.
4. After finishing writing, I reflect on my essay holistically to see if it achieves the goals of the writing task.
5. After finishing writing, I reflect on the composing process of "compose and revise, revise and compose".
